# Hormones and Vestibular Disorders: The Quest for Biomarkers

**DOI:** 10.3390/brainsci12050592

**Published:** 2022-05-02

**Authors:** Rhizlane El Khiati, Brahim Tighilet, Stephane Besnard, Christian Chabbert

**Affiliations:** 1 Team Pathophysiology and Therapy of Vestibular Disorders, Laboratory of Cognitive Neurosciences, UMR7291, Aix Marseille University-CNRS, CEDEX 07, 13007 Marseille, France; ghizlaineelkhiati@gmail.com (R.E.K.); brahim.thigilet@univ-amu.fr (B.T.); stephane.besnard@unicaen.fr (S.B.); 2 Research Group on Vestibular Pathophysiology, Unit GDR2074 CNRS, Aix Marseille University-CNRS, CEDEX 07, 13284 Marseille, France

**Keywords:** biomarkers, dizziness, hormones, Ménière’s disease, vestibular neuritis

## Abstract

The vestibular system exerts control over various functions through neural pathways that are not yet fully mapped. Functional dysregulations or tissue lesions at different levels of the peripheral and the central vestibular networks can alter these different functions, causing a wide variety of symptoms, ranging from posturo-locomotor alterations to psychiatric syndromes such as PPPD, including the deregulation of the main biological functions. These different symptoms differ by their expression kinetics (they each appear and regress with their own kinetics) by the targets affected (muscles, organs, and brain areas) and by the sensitivity specific to each individual. Vestibular pathologies thus cover a mosaic of distinct effects, and they involve various effectors—which constitute the many markers of their different types and stages. It is therefore crucial, to predict the onset of a vertigo syndrome, to follow its temporal course, or to monitor the impact of therapeutic approaches, and to have specific and reliable biomarkers. Hormonal variations are among the possible sources of biomarkers for neurotology. We know that specific hormonal profiles can promote the appearance of vestibular disorders. We also know that the expression of vertigo syndrome is accompanied by measurable hormonal variations. The link between endocrine deregulation and vestibular alterations therefore no longer needs to be proven. However, there are still few data on their precise correlations with the vertigo syndrome. This study was undertaken with the aim to deliver an extensive review of the hormonal alterations linked to vestibular disorders. A review of the literature covering the last two decades was carried out using the MEDLINE and COCHRANE databases in order to identify studies associating the terms vestibular system or vestibular pathologies and hormones. Bibliographic data provides several outcomes in terms of therapeutic innovation in the diagnosis and therapeutic follow-up of vestibular pathologies.

## 1. Vestibular Impairment: A Mosaic of Functional Alterations and as Many Potential Biomarkers

**Figure 1:**
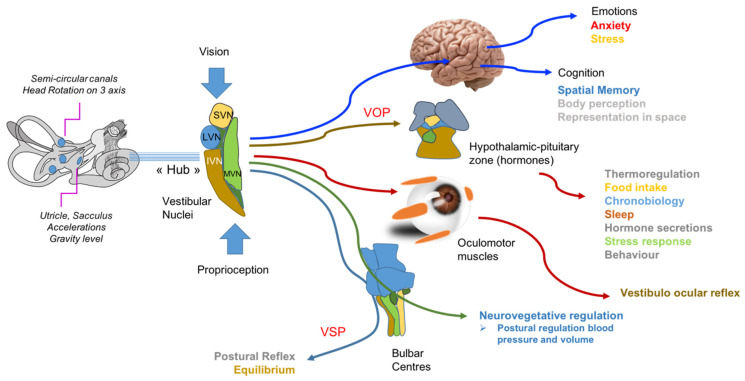
Biomarkers at the different levels of vestibular sensory information integration. VSP: Vestibulo Spinal Pathway; VOP: Vestibulo Ocular Pathway. SVN: Superior Vestibular Nuclei; MVN: Medial Vestibular Nuclei; LVN: Lateral Vestibular Nuclei; IVN: Inferior Vestibular Nuclei: Courtesy Dr S Besnard.

The vestibular system impacts different functions through different anatomical pathways (Figure 1). The vestibulo-spinal pathway, the first listed following the work of Flourens on the pigeon in 1830, ensures through descending bundles of motoneurons the control of the trunk and the leg muscles, allowing real time reactions to any disturbance of our equilibrium through adapted postural and locomotor responses to avoid falling. Thirty years later, Vulpian was the first to use the word nystagmus, soon followed by neurologists who established the existence of the vestibulo-ocular reflex. This reflex through the continuous control of the occulomotor muscles when moving the head, makes it possible to fix the gaze on a chosen target (an interlocutor, the sidewalk on which we walk, the staircase which we descend). Barany used it as a basis for the establishment of the caloric test of the vestibule function (rewarded with a Nobel Prize in 1914) [1].

It is only from the 1990s that we started to obtain anatomical and functional evidence of pathways originating from the brainstem vestibular nuclei and projecting towards the bulbar centers and the hypothalamic–pituitary complex. Yates is one of the pioneers who documented the functional pathways between vegetative brainstem centers and vestibular nuclei neurovegetative regulation [2,3]. Thanks to their work on animal models placed in modified gravity, Fuller and Besnard paved the way for the demonstration of vestibular control over the regulation of chonobiological functions, such as sleep, thermoregulation, food intake, and bone renewal [4,5,6,7,8,9].

We had to wait until 2010 to see an accumulation of experimental confirmations of a direct link between vestibule and cognition, with experimental validation of the role of the vestibule in memory and spatial navigation as well as in bodily self-awareness [10,11]. Finally, the vestibulo–cortical pathway currently deepened with anatomical and functional evidence of a strong link between the vestibule and emotions, with the recent inclusion in the International Classification of Vestibular Disorders (ICVD) of a new syndrome, PPPD, combining postural and perceptual deficits and psychiatric alterations [12].

Functional dysregulations or tissue lesions on the peripheral and the central vestibular networks impact these different functions and result in a wide variety of symptoms ranging from posturo-locomotor alterations to psychiatric syndromes such as PPPD (see other articles in this special issue). These different symptoms differ in their kinetics of expression (they each appear and regress with their own kinetics) in the organs they affect (muscles, organs, and areas of the brain) and sensitivity specific to each individual. These different symptoms thus cover a mosaic of effects and effectors which constitute as many biomarkers, characterizing the different types and stages of vestibular pathologies. It is therefore crucial to predict the onset of a vertigo syndrome, to follow its temporal course, or to monitor the impact of therapeutic approaches, and to have specific and reliable biomarkers.

## 2. Vestibular Disorders and Hormonal Dysregulation: An Obvious but Still Poorly Documented Link

A number of observations reveal clear links between the endocrine and the vestibular systems. Biological or histological analyses carried out in mammals have shown that different hormones are present in vestibular tissues. Hormones such as aldosterone, testosterone, progesterone, or estradiol have been found in the epithelial cells of the endolymphatic sac in mammals [13,14,15]. Vasopressin, through its action on transepithelial exchanges between perilymph and endolymph, is believed be a key player in the generation of hydrops associated with Ménière’s disease [16,17,18,19,20]. It has also been suggested as an inductor of motion sickness [21,22,23]. Furthermore, stimulation of the vestibular pathways has been shown to activate the central secretory nuclei, which in turn triggers endocrine variations [24,25,26]. Thus, stimulation of the vestibular pathways induces responses from the paraventricular nucleus (PVN) and the supraoptic nucleus [22,24,26,27]. The latter secrete vasopressin. Thyrotropin-releasing hormone (TRH) neurons in the paraventricular hypothalamic nuclei (PVN) also play a critical role in the function of the hypothalamic–pituitary-thyroid axis, as they stimulate TSH secretion [26]. Steroids are known to be directly involved in both the facilitative and deleterious effects of stress on vestibular compensation [28]. Vestibular stimulation also activates the sympathetic system [29] and the hypothalamic–pituitary axis [30], with modulation of CRH, ACTH, LHRH, and LH secretions [31]. The vestibular system sends projections to the suprachiasmatic nuclei (SCN) and the raphe [32] involved in the secretion of melatonin [26,33] and serotonin [34] as well as a projection bidirectional with orexinergic neurons [35,36]. The vestibular organs receive feedback from melatonin neurons [37]. Furthermore, receptors for different hormones such as vasopressin, cortisol, adrenaline, insulin, or sex hormones (testosterone, progesterone, and estrogen) [14,16,38,39,40,41,42] are expressed in the inner ear sensory organs and all along the nervous tract that projects to the brainstem vestibular nuclei [40]. The conditions for a modulating action of these hormones on the vestibular sensors and at the level of the vestibular sensory information integration zones are thus met. However, the molecular mechanisms supporting these actions, as well as their consequences on the vestibular function remain to be determined.

In order to determine whether the different phases of the vertigo syndrome are correlated with variations in the blood level of hormones, several clinical teams have compared blood samples taken at the time of the acute attack of peripheral vestibulopathies such as Ménière’s disease or BPPV and at a distance from them. These studies have demonstrated that vasopressin [31,43], cortisol [44,45], or parathormone [46] levels are significantly increased at the time of an acute vertigo attack in patients with Ménière’s disease and BPPV, respectively. These first data suggest that blood variations in circulating hormones could represent biomarkers of the different phases of the vertigo syndrome, with the potential opportunity to discriminate between the different types of vestibular pathologies.

Several epidemiological studies reveal that certain physiological states displaying particular hormonal profiles are especially exposed to vestibular disorders. This is the case in diabetes [47,48,49,50,51,52,53] and in thyroid pathologies [54,55,56]. Benign paroxysmal positional vertigo (BPPV) is predominant in postmenopausal women [57,58,59,60,61,62,63,64,65,66,67], while vestibular migraines may be linked to hormonal alterations [68,69]. If we can multiply the examples of comorbidity between specific hormonal profiles and vestibular disorders, the precise role of circulating hormones as triggers, accompanying factors, or reactive mechanisms to vestibular disorders remains to be determined. Together, these structural, functional, and epidemiological observations suggest a clear link between the hormonal and the vestibular spheres.

## 3. Hormonal Dysregulations as Biomarkers of the Different Types and Stages of Vestibular Disorders

The principle of a biomarker is to constitute a specific signature of a particular physiological or pathological state. To be considered as a biomarker, a single hormonal variation, or coordinated hormonal variations must occur only at a precise stage, or for a specific type of vestibular pathology. Such an event must be reproduced identically in the same individual, when the same physiological or pathological conditions are repeated; and, of course, it must be interindividually transposable. Taking the specific characteristics of each type of vestibular disorder, such as the etiology, neural circuits involved, organs and modulated cerebral zones, and time course, it is possible to conceive that a pattern of hormonal variations could represent a reproducible signature comparable to a biomarker. By definition, a biomarker must be measurable, quantifiable in a reliable way, and with standards in terms of methodology and norms. Hormone assays are routinely used in our disease areas to monitor the blood concentration of sex, thyroid, and adrenal hormones. Most biomarkers routinely measured in clinics for diagnosis or follow-up come from blood samples. Chemoluminescence immunoassay techniques are generally used as they are suitable for routine use on analyzers. Some hormones such as estradiol can still be measured by radioimmunology because they are more sensitive, especially in children. Hormone dosage is not a common practice.

## 4. Conclusions

Hormone dosage is not a common practice in the current diagnosis of vestibular patients. However, it could become a standard of care whether suitable protocols are established according to individual particularities, conditions of access to the patient, and the assay methods available in hospitals. Multicenter, double-blind clinical studies are now urgently needed to identify the different types of hormones, whose plasma or salivary levels may be altered during the different types of vestibular pathologies. Establishing controlled sampling and dosage protocols will open up new avenues for the development of new diagnostic tools and the therapeutic monitoring of vestibular pathologies.

## Data Availability

Not applicable.
